# Minimal Change Nephrotic Syndrome with Acute Kidney Injury after the Administration of Pfizer-BioNTech COVID-19 Vaccine

**DOI:** 10.1155/2023/5122228

**Published:** 2023-02-22

**Authors:** Erdinc Gulumsek, Dilan Damla Ozturk, Huseyin Ali Ozturk, Tayyibe Saler, Kivilcim Eren Erdogan, Ahmed Muhammad Bashir, Hilmi Erdem Sumbul

**Affiliations:** ^1^Department of Gastroenterology, University of Health Sciences-Adana Health Practice and Research Center, Adana, Turkey; ^2^Department of Internal Medicine, University of Health Sciences-Adana Health Practice and Research Center, Adana, Turkey; ^3^Department of Pathology, Cukurova University Faculty of Medicine, Adana, Turkey; ^4^Department of Internal Medicine, University of Health Science, Mogadishu Somali Turkey Training and Research Hospital, Mogadishu, Somalia

## Abstract

Nephrotic syndrome progresses with various metabolic disturbances, such as proteinuria over 3.5 grams in 24 hours, hypoalbuminemia, and hypercoagulability. Patients usually complain about diffuse edema throughout the body, which is secondary to hypoalbuminemia. It has many primary and secondary causes. Patients may require a renal biopsy to confirm the diagnosis. Besides, many secondary causes of nephrotic syndrome should be examined and excluded. Although many vaccines were developed due to the COVID-19, many side effects are still reported because of the Pfizer-BioNTech COVID-19 vaccine (COVID-19 mRNA and BNT162b2), which is widely used in Turkey. This study examines a case of nephrotic syndrome with acute renal injury after Pfizer-BioNTech vaccine.

## 1. Introduction

Nephrotic syndrome is a condition whose clinical picture is dominated by edema with the progression of metabolic disturbances such as proteinuria over 3.5 grams in 24 hours, hypoalbuminemia, and hypercoagulability [1]. Even though the most common cause of nephrotic syndrome in adults is diabetes mellitus, secondary causes such as amyloidosis, diabetes mellitus, systemic lupus erythematosus, mixed cryoglobulinemia, infections, drugs, and malignant diseases can also be observed in the cases in addition to primary causes such as minimal change disease, focal segmental, glomerulosclerosis, membranous nephropathy, and membranoproliferative glomerulonephritis [2, 3]. Renal biopsy may be required to determine the etiology in the presence of the new-onset idiopathic nephrotic syndrome. Diagnosing nephrotic syndrome without losing time and starting its treatment is vital [3]. While COVID-19 continues to spread worldwide, vaccines are the most effective ways to stop the pandemic. The Pfizer-BioNTech vaccine is also a genetic molecule-based vaccine in the form of mRNA developed against the COVID-19 virus [4]. The most common side effects of the vaccine are mild or moderate tenderness at the injection site, fever, fatigue, body aches, and headache. Still, rare side effects continue to occur as millions of people worldwide receive the Pfizer-BioNTech vaccine [5, 6]. In the related literature, a limited number of cases that developed minimal change disease have been reported after the Pfizer-BioNTech vaccine [7–11].

Antibody-dependent enhancement (ADE), in which SARS-CoV-2 is allowed to enter cells not expressing the angiotensin-converting enzyme 2 (ACE2) receptor via Fc-mediated attachment in the presence of binding, nonneutralizing antibody (non-NAb), or vaccine-associated enhanced respiratory disease (VAERD), in which immune complexes are deposited or “aberrant” T cell responses occur in the lungs of individuals who have received the vaccine, were proposed as possible mechanisms for the VAERD has typically been linked to the development of *T* helper 2 cell (TH2 cell)-biased CD4+ T cell responses, with significantly increased levels of interleukin-4 (IL-4), IL-5, and/or IL-13. This has been observed in animal models of infections with other coronaviruses as well as for previous whole-cell inactivated vaccines developed for respiratory syncytial virus and measles virus [8]. This study reports that glomerular disease is one of the rare side effects of this vaccine.

## 2. Case

A 58-year-old male patient, known case of diabetes mellitus, presented to the outpatient department with complaints of edema in the hands, feet, and around the eyes for about one month and a decrease in the frequency of urination. The patient had no history of using herbal products, smoking, or alcohol. The abdomen was soft, and no defensive rebound was found in the physical examination. Edema was present in the pretibial, periorbital, and sacral regions. He had rales at the bases of both lungs on auscultation. There was no palpable lymphadenopathy. The patient's laboratory tests are summarized in Table 1. He was admitted to our ward with a prediagnosis of acute renal injury and nephrotic syndrome.

According to the detailed history taken after the patient was admitted to the ward, he received the 1st dose of the Pfizer-BioNTech COVID-19 vaccine about 50 days ago and began to develop edema after the vaccination. He had the 2nd dose of Pfizer-BioNTech COVID-19 vaccine a week ago. Therefore, he presented to the outpatient clinic due to the progressive increase in his complaints after 2nd dose of vaccination. The patient was hypertensive in the ward follow-ups. Ultrafiltration treatment was carried out 7 times intermittently to the patient who had an allergic course with resistant hypertension and diuretic-resistant hypervolemia. As the patient had hypoalbuminemia in the laboratory tests and diffuse edema in the whole body in the physical examination, a 24-hour urine collection was planned for the patient. Proteinuria of 15,935 milligrams/day was detected in 24-hour urine. Thus, the nephrotic syndrome was considered, and tests were performed to exclude rheumatological diseases in the etiology of nephrotic syndrome. In the examinations performed, c3 and c4 levels were normal. ANA, c-ANCA, p-ANCA, anti-Scl-70, anti-Jo-1, anti-SS-A, anti-SS-B, anti-dsDNA, and anti-GBM results were negative. In the hepatitis serology of the patient, HbsAg, anti-HAV IgM, anti-Hbc IgM, anti-HCV, and anti-HIV were negative. The COVID-PCR test, performed twice with a 48-hour interval, was negative. No drug could cause nephrotic syndrome among the drugs he used, and there was no drug that he had just started. There was bilateral pleural effusion in the chest X-ray. No erythrocyte casts were detected in the urine sediment from the patient.

**Figure 1:**
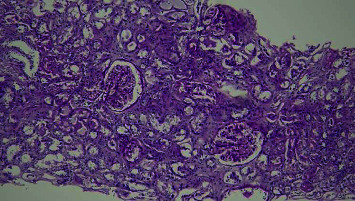
PAS ×100. Slight enlargement of the glomerular tangles and slight thickening of the basement membrane are observed.

**Figure 2:**
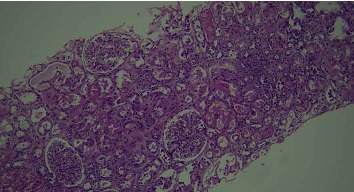
Slight enlargement of the glomerular tufts of hematoxylin cosine ×100 and slight thickening of the basement membrane are observed.

**Figure 3:**
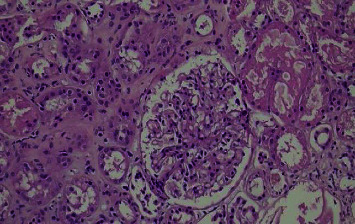
Slight enlargement of the glomerular tufts of hematoxylin cosine ×200 and slight thickening of the basement membrane are observed.

On the other hand, leukocyte and hyaline casts were noticed in the patient's urine. As a result, tuberculosis PCR and AFB were planned for sterile pyuria, but these tests were negative. The patient was examined for microvascular complications to exclude nephrotic syndrome due to diabetes mellitus and retinopathy was not detected in the examination. Peripheral smear, immunofixation electrophoresis, and protein electrophoresis were implemented to exclude multiple myeloma and other plasma cell dyscrasias because of the high sedimentation level, anemia, and nephrotic level proteinuria (while spot urine protein was negative by the dipstick method, 16 g/day proteinuria was found in 24-hour urine). No atypical cell and roll formation was detected in the peripheral smear. IgM kappa monoclonal gammopathy was detected in immunofixation electrophoresis. Abdominal ultrasound demonstrated no pathology, and superficial ultrasound did not detect lymphadenopathy. Bone marrow biopsy was performed, and the biopsy reported a hypercellular bone marrow and aspiration smear with increased myeloid and erythroid series. Flow cytometry sent from the bone marrow sample was not compatible with myeloma. As a result of the examinations, multiple myeloma and other plasma cell dyscrasias were excluded from the case. Considering the current clinical situation of the patient and the fact that monoclonal gammopathy detected in the bone marrow may also be observed in renal lymphoma, positron emission tomography (PET-CT) was carried out on the patient. No malignancy or additional pathology was suggested after the PET-CT findings. Cryoglobulins were tested to exclude the causes of secondary nephrotic syndrome, and it was negative. Rectal biopsy was performed to exclude amyloidosis in the patient with diffuse edema at the nephrotic level and biopsy was negative for amyloidosis. Lower extremity Doppler ultrasonography was performed because of edema in both lower extremities, and deep vein thrombosis was not found in the ultrasonography. A renal biopsy was planned for the patient to determine the cause of the newly developed nephrotic syndrome. Renal biopsy demonstrated that findings were consistent with minimal change disease (Figures 1–3).

We started 80 mg of methylprednisolone intravenously. Afterward, urea and creatinine values decreased, and urine output returned to normal. The dose was gradually reduced, and the patient continued methylprednisolone treatment. The clinical picture of hypervolemia improved on the 10th day after the treatment. The proteinuria decreased to 67 milligrams in the 24-hour urine collected as control samples. The patient is followed up with 16 mg methylprednisolone treatment. The history taken from the patient showed that his symptoms developed after the Pfizer-BioNTech COVID-19 vaccine. Hence, secondary causes of nephrotic syndrome were excluded with the examinations. The case was evaluated as a minimal change disease developed after the Pfizer-BioNTech COVID-19 vaccine, since the complaints in the history and the current clinic developed after the vaccine.

## 3. Discussion and Conclusion

The study presents a case that developed minimal change disease with acute renal injury after the Pfizer-BioNTech vaccine, an mRNA-based vaccine administered against the COVID-19 virus.

BNT162b2 is a nucleoside-modified mRNA vaccine that encodes the viral spike glycoprotein of SARS-CoV-2 formulated with a lipid nanoparticle [4]. Many complications have been reported after the Pfizer-BioNTech COVID-19 vaccine. The cases reported as postvaccine minimal change disease can be found in the literature [7–11]. The case of the study experienced nephrotic syndrome and severe acute renal failure. Renal biopsy was found compatible with minimal change disease. Nephrotic syndrome can have many causes [2, 3]. Primarily, although it is not possible to exclude all secondary causes definitively, the case of the study developed diffuse edema and acute kidney failure, and secondary causes were excluded after the diagnosis of nephrotic syndrome. As the clinic developed after the vaccine, the case was considered as a minimal change disease based on the Pfizer-BioNTech COVID-19 vaccine.

The first case of minimal change disease with acute renal injury after the COVID-19 vaccine was reported by Lebedev et al. [7]. In this case, the patient's complaints developed on the 4th day after the 1st dose of the Pfizer-BioNTech vaccine, and the 2nd dose of vaccine was not administered to the patient. The second case of minimal change disease with acute renal injury after the Pfizer-BioNTech vaccine was reported by D'Agati et al., and the symptoms in this case developed in the 1st week after the 1st dose of vaccination [9]. In both the cases, symptoms developed after the 1st dose of the Pfizer-BioNTech vaccine, and steroid treatment was given to the patients. The patients did not need ultrafiltration, and their clinical and laboratory findings improved with steroid treatment. In both cases, the studies do not involve an approach regarding the second dose of the vaccine.

On the contrary, in the case of this study, a nephrotic syndrome clinic was developed after one dose of Pfizer-BioNTech vaccine, and the patient did not receive any treatment during this time and his complaints increased progressively after the second dose of the vaccine. Unlike both the cases, the patient of the study needed repetitive ultrafiltration. The administration of the second dose of vaccine should be discussed in patients with complaints after the 1st dose of vaccination.

In conclusion, patients who develop diffuse edema after Pfizer-BioNTech COVID-19 vaccine should be monitored carefully. Blood pressure measurements, kidney function tests, and proteinuria should be followed up in these patients, and it should be considered that nephrotic syndrome may develop in these patients. In patients who develop postvaccine nephrotic syndrome with acute renal injury, it is vital to perform a kidney biopsy and to initiate corticosteroid therapy without delay if minimal change disease is diagnosed [10, 12–14].

## Figures and Tables

**Table 1:** Laboratory parameters of the patient before the treatment (admission to the hospital and 1 week after the 2nd vaccination) and after the treatment.

Laboratory parameters	Before the treatment	After the treatment
Urea (mg/dL)	91	35
Creatinine (mg/dL)	2.4	0.64
Sodium (mmol/L)	134	141
Potassium (mmol/L)	4.66	4.67
Blood gas pH	7.36	7.40
Serum albumin (g/dL)	1.9	2
Total protein (g/dL)	5	5.5
24-hour urine protein (mg/day)	15935	67
Alanine aminotransferase (U/L)	14	31
Aspartate aminotransferase (U/L)	27	49
WBC (/mm^3^)	9400	6500
Hemoglobin (g/dL)	11.2	11.4
Platelet (/mm^3^)	314000	310000
CRP (mg/L)	108.0	5.41
LDL (mg/dL)	248	284
Triglyceride (mg/dL)	336	334
Erythrocyte sedimentation rate (mm/h)	72	20

## Data Availability

The data and materials used to support the findings of the study are available from the corresponding author upon request.
